# Effects of yoga interventions on Anti-Müllerian hormone, androgen levels, and metabolic parameters in women with polycystic ovary syndrome: a systematic review

**DOI:** 10.1186/s12906-026-05313-6

**Published:** 2026-03-04

**Authors:** Shalini Chauhan, Taulant Muka, Sachal Sadiq Najaf, Ann Mary Babu, Leman Atmaca, Viktória Prémusz, István Karsai

**Affiliations:** 1 Faculty of Health Sciences, Doctoral School of Health Sciences, University of Pécs, Pécs, H-7621 Hungary; 2 Physical Education and Exercise Center, Medical School, University of Pécs, Szigeti út 12, Pécs, H-7624 Hungary; 3 GSY, Goodbye Stress With Yoga Project, University of Pécs, Pécs, H-7621 Hungary; 4 Evidence Based Medicine, Epistudia, Bern, 3008 Switzerland; 5 Research Department, Swiss TCM University, Bad Zurzach, Aargau 5330 Switzerland; 6 Department of Social and Political Sciences, Università degli Studi di Milano, Via Conservatorio 7, Milan, MI 20122 Italia; 7 National Laboratory on Human Reproduction, University of Pécs, Pécs, H-7624 Hungary; 8 Faculty of Health Sciences, Institute of Physiotherapy and Sports Science, University of Pécs, Pécs, H-7621 Hungary

**Keywords:** Yoga, Anti-Müllerian hormone (AMH), Androgen level, Female, Polycystic ovarian syndrome

## Abstract

**Introduction:**

Polycystic Ovary Syndrome is among the most prevalent endocrine disorders in women. Yoga has been suggested to affect hormonal and metabolic pathways, with implications for PCOS. To systematically review the effect of yoga on Anti-Müllerian hormone (AMH), androgen level, and metabolic parameters in women with PCOS.

**Methods:**

The search was carried out in Scopus, Embase, PubMed, Web of Science, Cochrane Trials, and Clinical Trials to identify randomized controlled trials (RCTs). Females with confirmed diagnoses (Rotterdam criteria), undergone yoga intervention were included to explore its effects on AMH, androgen levels, and metabolic parameters. The Revised Cochrane risk-of-bias tool for randomized trials (RoB 2) was used to assess the risk of bias. Due to the limited number of included studies and data heterogeneity, a meta-analysis was not performed; However, descriptive summaries of included studies are presented.

**Results:**

Five publications were included; however, three were identified as linked reports from a single clinical trial. Consequently, the review represents data from 258 unique participants. Only one reported result for AMH level with a mean difference and (95% CI) of changes of -2.03 ng/mL (-4.08 to 0.02), testosterone level, -8.61 ng/dL (-21.80 to 4.58), LH level -7.10 mIU/mL (-12.26 to -1.93), FSH 0.08 mIU/mL (-1.52 to 1.36). Two studies showed a mean difference and (95% CI) of changes for FBG level -4.50 mg/dL (-6.61 to -2.39) and -4.90 mg/dL (-12.34 to 2.54). Three studies showed a low risk of bias, one study had a moderate risk, and one exhibited a high risk of bias.

**Conclusion:**

The present review suggests that yoga may influence AMH level, androgen level, and insulin levels in PCOS; the evidence is limited, but it is still recommended to have robust RCTs on the long-term effect of yoga on PCOS. The visual presentation of the current systematic review is presented in Fig. 3.

**Trial registration:**

PROSPERO Trial Registration number: ID: CRD42022342913 (10/07/2022).

**Supplementary Information:**

The online version contains supplementary material available at 10.1186/s12906-026-05313-6.

## Introduction

Globally, 5- 9.2% % of women are affected by polycystic ovary syndrome (PCOS), and approximately 70% of women with PCOS remain undiagnosed worldwide [1].

PCOS is characterized by hormonal dysregulation [2–4], like elevated androgen levels and increased insulin resistance, affecting women`s health and quality of life [2]. Long-term effects of PCOS are also being recognized, such as increased risk of hypertension, diabetes mellitus, and cardiovascular disease [5, 6].

The Anti-Müllerian hormone may also play an essential role in the pathophysiology of PCOS through the regulation of folliculogenesis [7, 8]. Women with PCOS often have insufficient levels of Follicle-Stimulating Hormone (FSH), which leads to impaired follicular development [9]. Additionally, they have increased levels of Luteinizing Hormone (LH), which enhances ovarian androgen production [9].

Understanding lifestyle factors that could improve the management of PCOS is important. Yoga, classified as a combined mind–body intervention that integrates all the components of an individual’s lifestyle, can foster overall well-being [10–12]. Yoga is one of the best complementary medicines as it helps relax the body and mild, relieves stress, helps in weight loss, regulates blood circulation, and boosts the metabolism of the body [12, 13]. It is also proven that one of the very effective anxiolytic tools is yoga [14, 15].

The reducing stress effects of yoga are influenced to modulate the hypothalamic–pituitary–adrenal (HPA) axis activity, thereby reducing cortisol level, which is a chronic stress hormone [16, 17]. Increased levels of chronic stress are associated with hormonal disruption, which is also linked to increased Luteinizing Hormone (LH) and ovarian hyperandrogenism, which directly increases the production and signalling of Anti-Müllerian Hormone (AMH) by small follicles [18]. Yoga intervention can foster neuroendocrine balance and attenuate the stress response, it provides a non-pharmacological mechanism to normalize AMH and androgen levels in women with PCOS [13, 19].

**Fig. 1:**
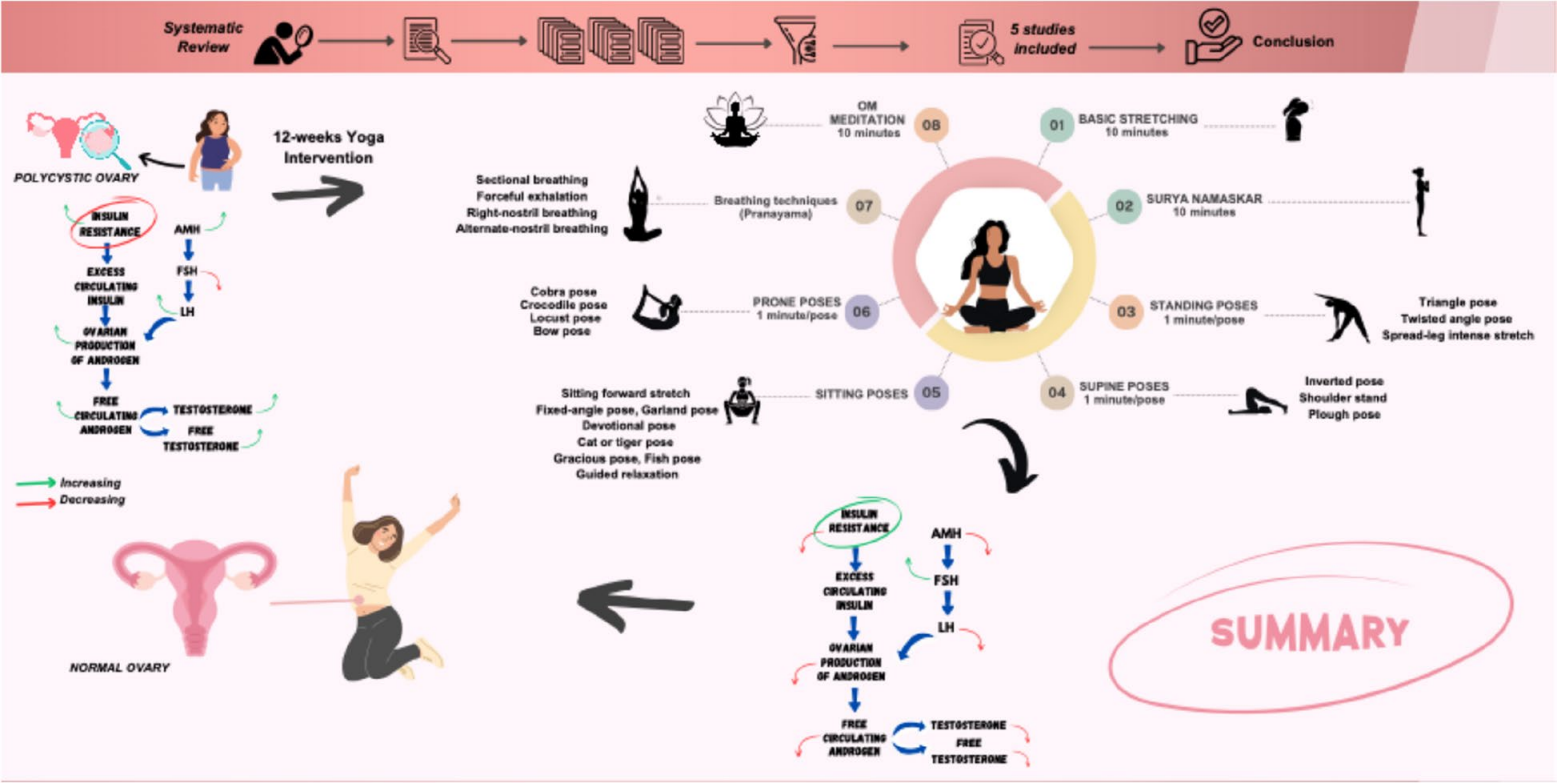
Visual abstract of the finding of the effect of yoga on AMH and androgen levels in females with PCOS

Evidence shows that consistent yoga practice not only promotes and maintains health conditions but also manages disease [20]. Many studies have been conducted that examine the effect of yoga therapy on anthropometric, neuroendocrine, and quality of life factors in women with PCOS [21–23]. However growing number of systematic reviews have assessed general physical activity and lifestyle changes for PCOS, which broadly covers general fertility and clinical symptoms or such as menstrual frequency, quality of life, etc. [24–26]. To the author’s best knowledge, there has been no systematic review conducted that exclusively and rigorously focused on the AMH levels, androgen levels, and metabolic parameters in women with PCOS. The novelty of the current review lies in its focused and systematic approach to specific biomarkers, which provides a targeted summary of structured yoga interventions biological effects. The comparison of the relevant prior’s systematic review versus the scope of this review is presented in Table 1 and Fig. 1.

**Table 1:** Comparison the summary of existing systematic reviews on yoga interventions with current systematic review

Reference	Primary Intervention Focus	Key Outcomes Examined (Scope)	Specific Focus on AMH, Full Androgen Panel, and Detailed Metabolism
Thakur et al. [27]	Yoga Intervention	Comprehensive: Blood Lipids, Glucose Metabolism, Endocrine Parameters, Quality of Life, Cardiovascular, Mental Health, BMI	No (Broad focus; lacked exclusive synthesis of the specific triad: AMH, Detailed Lipids)
Verma et al. [28]	Yoga Intervention	Population: Postmenarchal/Premenopausal Females; Outcomes:Menstrual Irregularity, Clinical Hyperandrogenism, Fasting Glucose, Fasting Insulin, HOMA-IR	No (Focus is on select metabolic indices; lacks AMH, and comprehensive lipid profile)
Current Systematic Review	Yoga Intervention	AMH, Total/Free Testosterone, Fasting Insulin, Lipid Profile	Yes (Exclusive and Primary Focus)

*Abbreviations*: *AMH* Anti-Müllerian Hormone, *HOMA-IR* Homeostatic Model Assessment of Insulin Resistance, *BMI* Body Mass Index

## Methods

This systematic review was conducted following two recent guidelines [29, 30] and reported adhering to the Preferred Reporting Items for Systematic Reviews (PRISMA) checklist [31]. The review protocol is registered in PROSPERO (ID: CRD42022342913).

### Data source search strategy and study selection

Six bibliographic databases were searched for relevant studies, including PubMed, Cochrane CENTRAL, Embase, Web of Science, Scopus, and Clinical Trials. The search was conducted up to 21/01/2024. From the eligible articles, reference lists were also checked. Additionally, a search in the Google engine was performed to find checking references and grey literature, including theses and conference abstracts.

The selection of the studies was performed by two authors, the first author (SC) and the third author (SSN). Both authors worked in parallel and selected the studies that were eligible for our selection criteria by the title and abstract screening using Rayyan. Disagreements between the two authors regarding including studies were resolved by discussion at each step, or if needed, the last author (VP) was involved in discussing the study selection and decided on the final study selection. Similarly, the full-text screening was performed in parallel with two reviewers.

### Eligibility criteria

Studies were included if were randomized controlled trials; (ii) included women with PCOS (Rotterdam criteria); (iii) assessed the effect of yoga, and explored outcomes Anti-Müllerian hormone (AMH), and Total Testosterone level, Luteinizing Hormone (LH) and Follicle-Stimulating Hormone (FSH), Fasting Insulin (FI), Fasting Blood Glucose (FBG), Systolic and Diastolic Blood pressure (SBP and DBP) and Weight loss. A summary of the inclusion and exclusion criteria based on the PICO (population, intervention, comparison, and outcome) framework is provided in Table 2.

**Table 2:** Criteria for inclusion/exclusion based on PICO framework

	**Inclusion criteria**	**Exclusion criteria**	**PubMed Search Strategy (MeSH terms and keywords)**
Population	Female with confirmed diagnosis of PCOS (Rotterdam Criteria)	Female with uncertain or undiagnosed PCOS status. Female who had undergone any surgical intervention for example cystectomy, ovarian drilling related to PCOS.	Polycystic Ovary Syndrome [MeSH] PCOS [Title/Abstract] Oligomenorrhea Hyperandrogenism [MeSH] Stein Leventhal Syndrome [Title/Abstract]
Intervention	All yoga form was included. Studies in which yoga therapy as an intervention was eligible for the review. No limitation to the timeline of Intervention given.	Studies with combined intervention with yoga for example yoga combined with other form of physical activity, diet, or other therapies.	Yoga [MeSH] Ashtanga-based Yoga Therapy (AYT) [Title/Abstract] Bikram Yoga [Title/Abstract] Hatha Yoga [Title/Abstract] meditation [MeSH] Kundalini Yoga [Title/Abstract] Yoga Kundalini [Title/Abstract] Iyengar Yoga [Title/Abstract] Isha kriya [Title/Abstract] Sudarshan Kriya yoga [Title/Abstract]
Comparison	Intervention group compared to the control group which doesn’t receive any kind of therapy, which means control group with standard care and without any treatment or intervention. (If trial includes multi-arms, we included any arm that meets the review inclusion criteria).	NA	
Outcome	Anti-Müllerian Hormone (AMH), Luteinizing Hormone (LH), Follicle Stimulating Hormone (FSH), Reproductive hormone and Sexual hormone. Systolic Blood Pressure and Diastolic Blood Pressure fasting blood glucose, fasting insulin, and postprandial glucose (PPG) test weight, BMI, waist circumference and hip circumference.	No reporting or partially reporting outcome.	
Study Design	Randomized control trial	All studies which are not randomized, or which cannot meet fundamental characteristics of RCT.	Randomized controlled trial [MeSH] Controlled clinical trial [MeSH] Clinical trial [MeSH]

*Abbreviations*: *PCOS* polycystic ovarian syndrome, *MeSH* Medical subject heading

### Data extraction process and quality assessment

A data extraction form was developed and applied, containing information on 1) author’s name, 2) year of study, 3) blinding, 4) allocation method, 5) sample size, 6) age of participants, 7) diagnostic criteria, 8) duration of intervention, 9) outcome reported, and 10) primary outcome. We used the Cochrane (RoB2) tool for the assessment of all included Randomized Controlled trials in accordance with the Cochrane Collaboration’s guidelines [32]. Risk of Bias in all the included studies was classified as Selection of the reported result, Randomization process, Deviations from the intended interventions, Missing outcome data, and Measurement of the outcome.

### Data synthesis

Data was synthesized from all the included studies, and a summary of outcomes was presented. This summary included the mean value of pre-intervention and post-intervention and the mean difference (intervention minus control group) reflecting the treatment effects. Where necessary, the conversion of units was performed to present the findings in SI units for comparability and consistency. The primary reason for not performing the meta-analyses was the limited number of included studies, as well as the substantial heterogeneity observed among the studies. Instead, we used a narrative synthesis approach.

## Results

### Studies identified

A total of 275 citations were identified after running the search in 6 databases (PubMed = 21, Embase = 45, Cochrane Trial = 54, Scopus = 83, Web of Science = 66, Clinical Trials = 6). No relevant studies were identified through a Google search. Of these, 183 citations were left after removing the duplicates using the referencing manager EndNote. After title and abstract screening, 31 relevant citations were considered for full text. Among these, 25 studies were excluded (3 citations were research protocols, 3 studies did not report the results, and 9 studies’ full text was not available (In this case, corresponding authors of these studies were contacted to have access to full-text studies but none responded), 3 studies used an intervention other than yoga, 1 study did not study the outcome of our interest, 3 studies used other study designs, and 3 studies did not include PCOS women), leaving 5 studies for our final analysis.

**Fig. 2:**
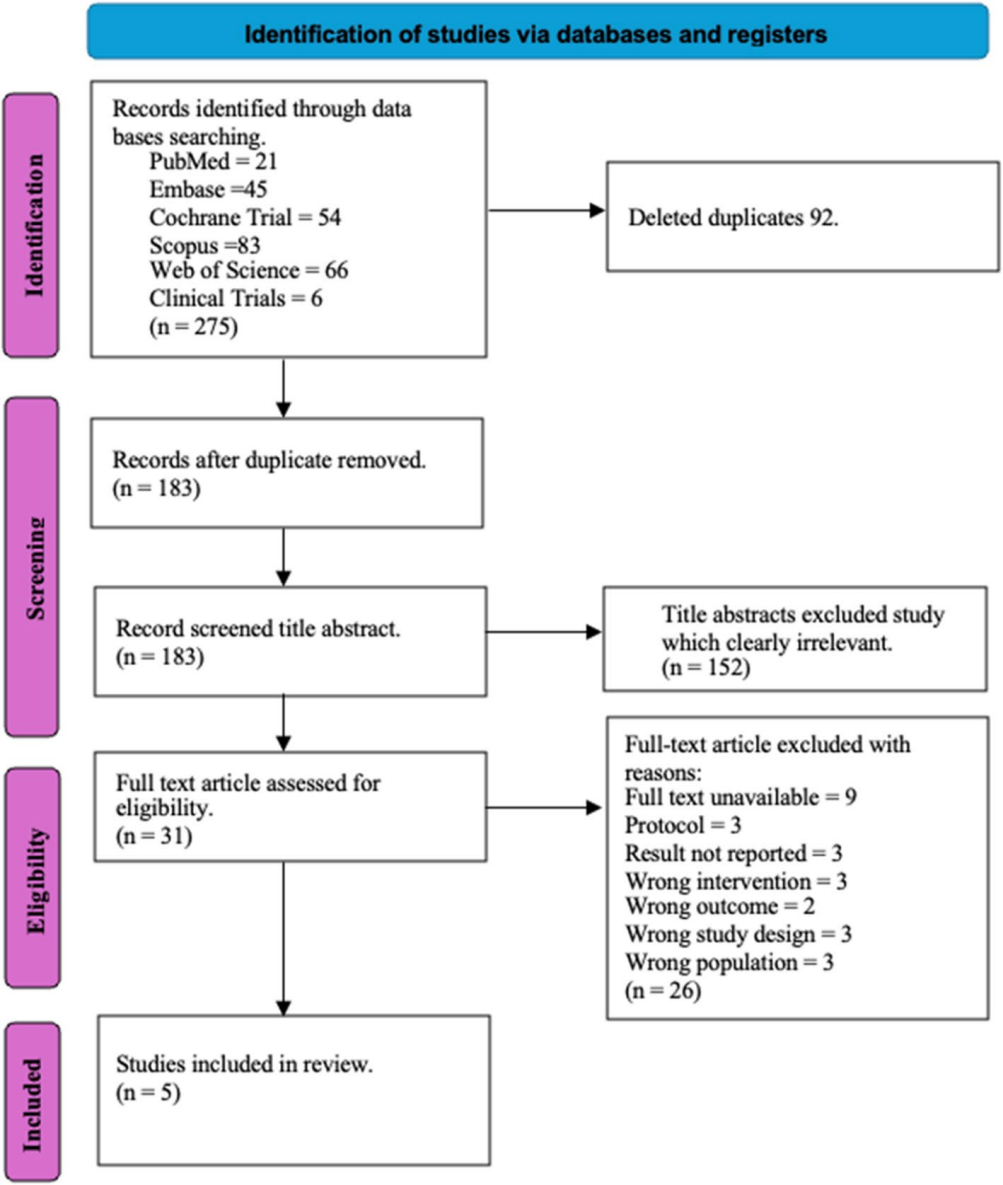
PRISMA 2020 flow diagram for new systematic reviews which included searches of databases and registers only

A visual representation of the study selection process is shown in the PRISMA flow chart (Fig. 2) [33]. An overview of the characteristics of the included studies has been presented in Table 3.

**Table 3:** Overview of the characteristics of included studies

**Reference**	**Year**	**Blinding**	**Allocation Method**	**Sample Size**	**Age (years)**	**Diagnostic Criteria**	**Duration of Intervention**		**Outcome Reported**	**Primary Outcome**
							Weeks	Time [22]		
Nidhi [34]	2013	Single Blinded	Computer Generated	90	15—18	Rotterdam criterion	12	60	FSH (follicle-stimulating hormone); LH, (luteinizing hormone); Prl (prolactin); TT, (total testosterone); AMH (Anti-Müllerian hormone)	AMH (Anti-Müllerian hormone)
Nidhi [35]	Mar2012	Single Blinded	Not Mentioned	85	15—18	Rotterdam criterion	12	60	FI (fasting Insulin); FBG (fasting blood glucose); H (hyperandrogenism); HC (hip circumference); Weight loss; HDL (High-density lipoprotein); HOMA-IR, (Homeostasis Model Assessment, insulin resistance); LDL (low-density lipoprotein); O, (oligomenorrhea); TC (total cholesterol); TRIG (triglycerides);	Glucose, lipid, and insulin values
Mohseni [22]	2021	Not Mentioned	Open Envelop	67		Rotterdam criterion	6	90	Systolic blood pressure, Diastolic blood pressure	Anthropometric
Patel [36]	2019	Blinded	Unique, Sequential Number	31	23- 42	Rotterdam criterion	12	60	FBG (Fasting Blood glucose); BMI (body mass index); HOMA-IR (homeostatic model assessment for insulin resistance); free testosterone:	Endocrine, Cardiometabolic, or psychological parameters
Nidhi [35]	Sep2012	Single Blinded	Computer Generated	90	15—18	Rotterdam Criteria	12	60	Emotions, body hair, weight, infertility, and menstrual problems	Quality of Life

Nidhi et al. [34, 35] refer to the same study population

### Studies and participants

A total of 5 studies were included in this review. However, a detailed analysis of the study settings and participant demographics revealed that three reports [23, 34, 35] originated from the same randomized controlled trial. To ensure methodological integrity and avoid the inflation of data, these were treated as linked publications. Consequently, the final analysis represents 258 unique participants. These featured 10 study arms comparing yoga interventions—either in isolation or combined with other lifestyle modifications—against control groups receiving usual care or conventional exercise. Control groups included 128 unique participants (conventional physical activity, routine care, or no intervention), while the intervention groups consisted of 130 unique participants. Geographically, three publications (representing one major trial) were conducted in India, while the other two studies were conducted in the USA and Iran.

### Interventional characteristics and quality assessment of included study

**Fig. 3:**
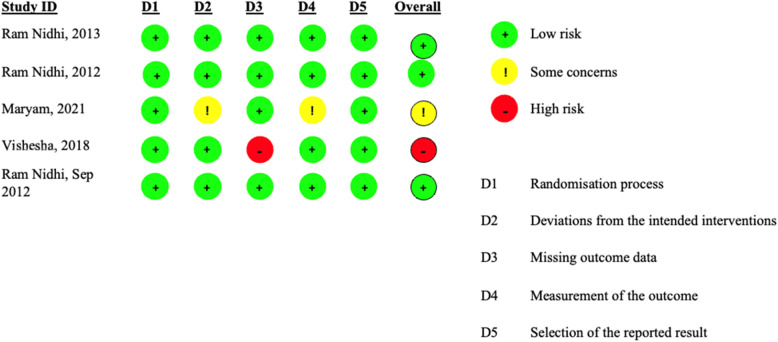
Risk of bias summary of the included randomized controlled trials (RCTs) according to the bias assessment tool of the Cochrane Collaboration (RoB 2)

Yoga intervention with Yogic lectures and counselling on the management of stress and yogic lifestyle was given in three studies [23, 34, 35]; one study included mindful yoga intervention, in which intervention is concluded with meditation and mindful “I am” statements [36]. While one study included yoga intervention alone without any combination [22]. All participants received training in yoga practice, but the frequency of the yoga session practice varied across studies. For example, one study had a yoga intervention for 6 weeks with 90 min/per session. Another study offered 12 weeks of yoga intervention, 3 times per week with 60-min sessions, and three studies provided 12 weeks of yoga intervention, once a week, 90-min sessions (Table 3). Three studies were judged at a low risk of bias [23, 34, 35] while one study was at risk of bias [36], and one study showed some concerns [22] (Fig. 3).

### Outcome measures

Primary outcome measures included AMH (*n* = 1), total testosterone (*n* = 1), free testosterone (*n* = 1), which was reported in one study, and LH and FSH (*n* = 1), Fasting Insulin (FI) (*n* = 2) and Fasting Blood Glucose (FBG) (*n* = 2), which was reported in one study. Secondary outcomes included, weight loss (*n* = 2), Systolic Blood Pressure, and Diastolic Blood Pressure (*n* = 1). There were no adverse effects documented in any of the included studies (Table 4). All the studies included in the analyses reported the baseline and endpoints. The findings of these included studies are presented in Table 5.

**Table 4:** Details of intervention and outcome reported in included studies

Study	Participants	Exercise Intervention	Outcome Changes significantly	Outcome changed non- significantly/unchanged
Nidhi [34]	90 PCOS (45/Per group) 15–18 years; mean 16.22; BMI mean 20.36; Rotterdam criteria	12 weeks (1 h/day) Yoga Intervention. The practices consisted of asanas. (yoga postures), pranayama, relaxation techniques, meditation, and lectures on yogic lifestyle and stress management through yogic counselling.	AMH↓,LH↓,Testosterone↓,	FSH↓, and Prolactin↓
Nidhi [35]	85 PCOS (42 Yoga group/43 control group) 15–18 year: mean age 16.22; BMI mean 20.30; Rotterdam Criteria	12 weeks (1 h/day) Yoga Intervention The practices consisted of asanas. (yoga postures), pranayama, relaxation techniques, meditation, and lectures on yogic lifestyle and stress, management through yogic counselling.	FI↓, FBG↓,TRIG↓,TCHL↓,LDL↓, VLDL↓,TC/HDL↓	HDL
Mohseni [22]	61 PCOS (31 Yoga group/30 control group) 15–18 year: mean age of yoga group 30.77, and mean age of control group 30.35; BMI mean of yoga group 25.96, mean age of control group 25.50; Rotterdam criteria	6 weeks Yoga intervention; twice a week 90-min yoga session by instructor:5 sessions per week at home by the participants themselves; 25 min of exercises in order to relieve physical stress, followed by 45 min of asana exercises, and the last 20 min were dedicated to deep relaxation exercises	Abdominal circumference ↓, Hip circumference ↓, Hirsutism (Ferriman-Gallwey) ↓	Systolic blood pressure, Diastolic blood pressure
Patel [36]	22 PCOS (13 Yoga group/9 control group) 22–43 year: mean age of yoga group 30.09, and mean age of control group 31.2; BMI mean of yoga group 35.1, mean age of control group 35.4; Rotterdam criteria	12 weeks Yoga intervention; 3 times per week 60-min yoga session by certified yoga; practice that included pranayama exercises (3-part yogic breath: ujjayi breath, alternate nostril breathing, and breath of fire), vinyasa flow yoga, restorative yoga asanas, and concluded with meditation that incorporated guided imagery of healing energy and mindful “I am” statements	Free T↓, DHEA↓, Adiponectin↓,	FBG↓,FI↓,DHEAS↓
Nidhi [35]	90 PCOS (45 Yoga group/45 control group) 15–18 year: mean age of yoga group 16.22, and mean age of control group 16.22; BMI mean of yoga group 20.30, mean age of control group 21.22; Rotterdam criteria	12 weeks (1 h/day) Yoga Intervention. The practices consisted of asanas (yoga postures), pranayama, relaxation techniques, meditation, and lectures on yogic lifestyle and stress management through yogic counselling.	Emotional disturbance↓, Body Hair↓, Weight, Menstrual problem↓	Infertility↓

*Abbreviations*: *PCOS* polycystic ovarian syndrome, *FI* fasting insulin, *FBG* fasting blood glucose, *AMH* Anti-Müllerian hormone, *LH* luteinizing hormone, *FSH* follicle-stimulating hormone, *TT* total testosterone, *DHEAS* dehydroepiandrosterone sulfate, *A4* androstenedione, *SBP* systolic blood pressure, *DBP* diastolic blood pressure; Serum lipids: *TC* total cholesterol, *TRIG* triglycerides, *HDL* high-density lipoprotein, *LDL* low-density lipoprotein, *VLDL* very low-density lipoprotein, TC/HDL. *Note: Nidhi et al. (March2012, Sep2012, 2013) refer to the same study population

**Table 5:** Reported outcomes in all included studies

Outcome	Study Reference	Intervention	Pre Mean ± SD	Post Mean ± SD	*p*- value^a^	Control Group	Pre Mean ± SD	Post Mean ± SD	*p*- value^b^	*p*-value^c^
AMH (ng/mL)	Nidhi [34]	Holistic Yoga Module	6.25 ± 3.79	3.73 ± 2.25	NR	Physical Exercises	4.79 ± 2.33	4.30 ± 2.88	NR	0.006
LH (mIU/mL)	Nidhi [34]	Holistic Yoga Module	11.94 ± 8.32	7.84 ± 6.13	NR	Physical Exercises	7.26 ± 5.18	10.26 ± 8.66	NR	0.005
FSH (mIU/mL)	Nidhi [34]	Holistic Yoga Module	5.97 ± 1.87	5.58 ± 1.90	NR	Physical Exercises	5.76 ± 2.50	5.45 ± 1.73	NR	0.474
TT (ng/dL)	Nidhi [34]	Holistic Yoga Module	39.55 ± 21.40	33.55 ± 19.93	NR	Physical Exercises	29.48 ± 15.27	32.09 ± 16.46	NR	0.014
FI (pmol/L)	Nidhi [35]	Yoga Practice	60.62 ± 23	51.58 ± 17.53	< 0.001	Conventional Physical Exercises	70.31 ± 33.09	81.4 ± 47.59	< 0.001	NR
FI (mIU/mL)	Patel [36]	Mindful Yoga Intervention group	mean range, 11.0 (2–33)	mean range 10.0(2–47)	0.824	Control Group	14.8 ± 3.9	12.7 ± 2.7	0.328	NR
Free T (pg/mL)	Patel [36]	Mindful Yoga Intervention group	5.96 ± 1.2	4.24 ± 0.6	0.413	Control Group	7.39 ± 1.61	7.36 ± 1.29	0.967	NR
Fasting BG (mg/dL)	Patel [36]	Mindful Yoga Intervention group	87.9 ± 1.9	86.3 ± 1.9	0.403	Control Group	89.2 ± 4.5	92.1 ± 2.7	0.387	NR
	Nidhi [35]	Yoga Practice	78.1 ± 18.01	73.87 ± 6.12	< 0.001	Conventional Physical Exercises	75.13 ± 5.76	75.8 ± 6.12	0.47	NR
SBP (mmHg)	Mohseni [22]	Yoga Exercise	110.23 ± 10.33	110.00 ± 10.26	0.938	Control Group	110.26 ± 10.15	110.29 ± 1.42	0.402	NR
DBP (mmHg)	Mohseni [22]	Yoga Exercise	60.73 ± 10.34	70.20 ± 0.89	0.096	Control Group	70.26 ± 10.06	70.48 ± 0.99	0.245	NR
Weight (kg) Weight (kg)	Nidhi [34]	Holistic Yoga Module	48.24 ± 6.53	48.28 ± 6.75	NR	Control group	51.83 ± 7.66	52.62 ± 6.97	NR	0.882
	Nidhi [35]	Holistic Yoga Module	4.44 ± 1.72	2.92 ± 1.53	NR	Control group	3.79 ± 1.48	3.27 ± 1.27	NR	0.018

*Abbreviations*: *SD* standard deviation, *NR* not reported. *Note: Nidhi et al. [34, 35] refer to the same study population

^a^Significant at *P* <.05—Intra group

^b^Significant at *P* <.05—Intra group

^c^Significant at *P* <.05—Inter group

### Primary outcomes

#### AMH and total testosterone level

Only one study reported on the AMH level [23]. This study showed a favorable result for the intervention in lowering AMH level and total testosterone levels through yoga intervention in women with PCOS compared to the physical exercise group, with a mean difference for AMH was −2.03 ng/mL (95% CI: −4.08 to 0.02), and for testosterone of −8.61 ng/dL (95% CI: −21.80 to 4.58).

One other study reported on free testosterone levels [23]. Comparing the yoga intervention to the control group, this study showed a moderate decrease in free testosterone level, with a mean difference was −1.69 pg/mL (95% CI: −2.57 to −0.80).

#### LH and FSH

In all included studies, there was only one study [23] that reported the LH and FSH levels. Based on this study, LH levels were lowered compared to the control group. The mean difference was −7.10 mIU/mL (95% CI: −12.26 to −1.93), demonstrating significant differences between the groups. The FSH level after the 3 months of yoga intervention showed a nonsignificant (*p* = 0.474) difference between the two intervention groups; the mean change after yoga intervention was 0.40, and the physical exercise change was 0.31. However, the mean difference between the yoga intervention and control group was −0.08 mIU/mL (95% CI: −1.52 to 1.36).

### Metabolic outcomes

#### Fasting Insulin (FI)

Two studies reported fasting insulin [34, 35]. The study showed no statistically significant difference reported before and after the yoga intervention (*p* = 0.328), due to the lack of information provided, it is not possible to calculate the mean difference between the groups. The second study [35] showed a significant reduction in fasting insulin after the yoga intervention, *p* < 0.001, with the mean difference of the yoga group compared to the control group being −20.11 pmol/L (95% CI: −43.28 to 3.02).

#### Fasting Blood Glucose (FBG)

FBG was reported in two included studies [35, 36]. There were no significant differences *p* = 0.403, after the 3 months of yoga intervention in this study [36]. This study showed ed mean difference and (95% CI) of changes in the yoga intervention group compared to the control group was −4.50 mg/dL (95% CI: −6.61 to −2.39). However, due to the small size and high risk of bias, it was suggested that the results of this study be interpreted with caution. The second study reported a significant reduction after the yoga intervention, *p* < 0.001 [35], with the mean difference of the yoga group compared to the control group being −4.90 mg/dL (95% CI: −12.34 to 2.54).

### Additional outcomes

#### Systolic and Diastolic Blood Pressure (SBP and DBP)

Only one study [22] reported SBP and DBP, and based on the findings, there were no significant differences reported in the two groups. and The mean differences for SBP and DBP of yoga intervention compared to the control group were −0.26 mmHg (95% CI: −6.61 to 6.09) and −9.25 mmHg (95% CI: 4.06 to 4.44). However, the study shows some concerns about the risk of bias and reports the limitation that the sample is small. Interpreting the result with caution is recommended.

#### Weight loss

Two studies [34, 35] reported weight loss comparing the weight before and after the intervention. There were no significant differences in the weight of individuals in one of the studies although this study showed a slight decrease in mean difference in the yoga group compared to the control group −1.00 kg (95% CI: −2.08 to 0.08), while in the other one, there were significant differences *p* = 0.018 in the weight of the intervention group with a slight decrease in the mean difference of the yoga group compared to the control group showing a slight decrease of −0.75 kg (95% CI: −5.75 to 4.25).

## Discussion

This current systematic review aimed to provide a comprehensive assessment of the evidence from RCTs on the effect of yoga intervention on androgen levels, AMH levels, and metabolic health parameters in women suffering from PCOS. Only 5 Studies were identified as eligible studies. A total of five publications were identified as eligible for this review. However, as three of these reports [23, 34, 35] were found to be linked publications originating from a single clinical trial, the final analysis represents 258 unique participants with PCOS. The studies have considerable clinical heterogeneity across a range of outcomes reported.

The studies have considerable clinical heterogeneity across a range of outcomes reported. Although our systematic search was conducted up to 21/01/2024 across six major databases, only 5 randomised controlled trials met our eligibility criteria, which were specifically designed to focus on high-quality data regarding AMH and androgen levels in PCOS women diagnosed by Rotterdam criteria**.** It is important to mention that none of the included studies in the current systematic review reported any side effects and that long-term effects remain unexplored. Furthermore, three of the included studies have a low risk of bias, while one has a high risk of bias, and one included study shows some concern (moderate risk of bias).

### Impact of yoga intervention on hormonal profiles associated with PCOS pathogenesis

There was only one included study [36] that reported the AMH level after the yoga intervention. It is also the first study, that analyses the effect of 3 months of yoga intervention on the AMH level. This study reported a significant reduction in AMH level (*p* = 0.006) after yoga intervention compared to physical exercise. This study does not only state the importance of yoga intervention in women with PCOS but also states that yoga therapy may provide better changes in AMH levels than physical exercise intervention.

A well-designed randomized control trial [37] of 26 weeks on lifestyle, diet, metformin, and also androgen suppression with Dexamethasone. This trial includes 50 women, and the result of this trial reported no significant change in the AMH level after the intervention. Also, the contradictory observation reported an increase in AMH level from 12.6 ng/mL to 14.1 ng/mL in the specific group that received Dexamethasone. Evidence from more than 10 years has consistently reported that AMH levels are 2–threefold elevated in patients with PCOS [38]. This shows that AMH level in PCOS women is an important factor to be addressed in studies with yoga as an intervention therapy.

Of the two included studies that reported free testosterone levels and total testosterone levels, it was shown that after 3 months of yoga intervention, testosterone levels in participants in yoga intervention (mean change = 6.01) significantly decreased than in physical exercise (mean change = 2.51) (*p* = 0.014). However, in the case of PCOS women, it is very common to find a high androgen level, but in this study, all the participants were well within the normal range of total testosterone (< 82 ng/dl). Another included study reported that free testosterone after 3 months of yoga intervention can significantly decrease the level of free testosterone (*p* < 0.04). Although this study has shown a high risk of bias, the results should be interpreted with caution.

Three-month yoga intervention can alter LH hormone levels [36]. The current included study showed a significant reduction in LH level in yoga intervention (mean change 4.09, *p* = 0.005), while the same study reported a very minor reduction in FSH level after yoga intervention (mean change 0.40, but not significantly *p* = 0.47). A similar result can be shown in the study on the effect of Metformin for 6 months in obese teenage girls [39]. This shows the necessity of yoga as a lifestyle intervention in women with PCOS. However, in the current included study, there was a noteworthy change shown after the yoga intervention. This could be due to nonuniformity in the duration of hormonal assessment in this study. A systematic review regarding the effect of exercise intervention on women having PCOS revealed that overall, the exercise intervention generally did not alter LH level and FSH levels in these women [40]. Specifically, the trial with aerobic intervention showed very limited effectiveness in altering the level of LH and FSH [38, 39]. It is very interesting to see that this review highlighted that the study with yoga intervention was lowering the level of LH and FSH levels. These findings recommended the further exploration of yoga intervention with a larger sample size in PCOS women for the management of hormonal imbalance.

Impaired glucose intolerance, along with hyperinsulinemia, is the most frequent factor found in women with PCOS [41, 42]. Although the actual factor responsible for PCOS is still unknown, insulin plays a very important role in the pathogenesis of PCOS manifestation [43]. Two studies were included that reported fasting blood glucose and fasting insulin levels. There was a significant reduction found in the FBG (mean change = 0.24 (mg/dL), significantly *p* < 0.001) and FI (mean change = 9.04 (pmol/L), significantly *p* < 0.001 the 3-month intervention in the participants by the yoga intervention in one of the studies [35]. It is important to note that this change has been reported in the population aged 15–18 years. However, to see the change in various age groups of women with PCOS, further studies are needed to clarify these findings. Another study that reported a difference in fasting blood glucose and fasting insulin after yoga intervention showed no significant differences in fasting glucose and fasting insulin after 3 months of yoga intervention. Although this study has a high risk of bias, it is advisable to interpret this finding with caution.

There were several trials on the other exercise interventions to see the impact on insulin and blood glucose levels. RCT trial with aerobic intervention yielded improvement in fasting blood glucose and fasting insulin levels [44]. This has been supported by other trials with the combination of PRT (progressive resistance training) also showed improvement in the fasting insulin levels [45], Although the studies with PRT intervention did not show a change in Fasting insulin [27, 46]. However, it is recommended the necessity of yoga intervention to elucidate the impact of yoga in PCOS women with varying BMI, ages, and metabolic health.

Only one included trial in the current systematic review reported the SBP level and DBP level. The finding of this trial showed that the 6 weeks of yoga intervention did not show significant effects on DBP (mean change = 10.53, *p* = 0.245) and SBP (mean change = 0.23, *p* = 0.402) [17]. However, the other study on the effect of yoga intervention in participants with cardiac and fibrillation demonstrated a significant change in systolic blood pressure and diastolic blood pressure [47]. The study of the effect of yoga trials on patients with hypertension showed that yoga intervention in the classroom does not show a significant reduction in patients with hypertension [48]. These contradictory findings lead to the understanding that the reason behind this inconsistency with the results can be due to the severity and duration of the intervention as well as the disease of the patients.

The non-uniform findings across primary and secondary outcomes highlight a major limitation: They demonstrated methodological and substantial clinical heterogeneity among the included studies. Specifically, there is significant variation in the yoga intervention protocols, with interventions ranging in duration from 6 to 12 weeks and 60 to 90 min per session. Additionally, the wide variation in the intervention’s specific components (e.g., asanas, pranayama, and meditation) makes it more challenging to identify the optimal protocol that can improve hormonal and metabolic outcomes in women with PCOS. This limitation should be addressed in future research to establish effective clinical guidelines. Furthermore, the publication bias is an important consideration in systematic reviews; standard statistical tests such as Egger’s test and funnel plots require a minimum of 10 studies to provide sufficient statistical power and avoid misleading results, as per the Cochrane Handbook for Systematic Reviews of Interventions [49]. Given that our review included only five studies, we followed a qualitative approach to minimize bias by searching multiple databases and clinical trial registries to identify all possible published and unpublished data.

A systematic review indicates that using different yogic limbs (asanas, pranayama, relaxation techniques, meditation, etc.) can help in managing polycystic ovarian syndrome with or without medications [27]. However, there was a high level of methodological bias in this systematic review, which makes it difficult to understand the effect of yoga on the different parameters that can benefit women with PCOS.

The need for a holistic approach to PCOS can be understood through another systematic review [46]. It is proven that women with PCOS suffer an increased level of menstrual irregularities, skin-related issues, and subfertility, and it is found that they suffer emotional well-being with an intermix of dysmorphia of the body [46, 47]. All these concerns can be screened and managed through a holistic approach provided by appropriate expertise [50]. It has also been proven that yoga offers a holistic approach to balancing the hormones of the body and aiding in the management of chronic health problems [13, 51].

The current systematic review highlights significant research limitations. Given that the increased AMH levels and androgen levels are key biomarkers [52], this research gap is crucial to address in future research. To address this limitation, there is a need to conduct well-designed Randomized controlled Trials with a large sample size, where AMH should be included as a primary or secondary outcome. Additionally, it has been shown that there is an urgent need for the standardized yoga protocol to clearly define the specific components, such as asanas, pranayama, and meditation, and consistently used across all studies, to generate strong and replicable evidence. Moreover, the included studies were conducted in India, which increases the possibilities of geographical bias. Long-term follow-up is required to determine the clinical relevance and durability of any observed AMH reduction and hormonal balance after standardized yoga intervention. Beyond AMH level, further research is also needed to confirm the effects of standardized yoga intervention on androgen levels and to clarify the long-term benefits on various metabolic parameters such as insulin resistance, lipid profiles, etc.

The current systematic review provides detailed information on the evidence showing how specific yoga interventions can affect maintaining androgen levels, AMH levels, and metabolic parameters among women with PCOS. Additionally, the current review also demonstrates the specific yoga protocol that could guide yoga instructors and researchers to build further RCT trials.

## Conclusions

Studies on yoga showed its potential to alter androgen, AMH, and insulin levels, albeit the evidence is limited to draw firm conclusions. In addition, the small number of participants included in the trials highlights the importance of more studies to generalize the result for all the women with different sociodemographic backgrounds.

The important recommendation that can arise from this systematic review is the necessity for well-methodologically designed RCT trials to evaluate the impact of yoga in managing PCOS focusing on AMH level, androgen, and insulin level. Additionally, it is recommended to build up trials to concentrate on the long-term effects of yoga and investigate the possible side effects among women of different ages and BMIs having PCOS; this can necessitate comprehensive reporting and standardized monitoring.

## Supplementary Information

Supplementary Material 1.

## Data Availability

The datasets used and/or analysed during the current study available from the corresponding author on reasonable request.
