# Supplementary material for: Effects of yoga interventions on Anti-Müllerian hormone, androgen levels, and metabolic parameters in women with polycystic ovary syndrome: a systematic review
Source: BMC Complement Med Ther. 2026 Mar 4;26:133. doi: 10.1186/s12906-026-05313-6 (PMC13067475; doi:10.1186/s12906-026-05313-6)
Supplement: Supplementary file 1 — Supplementary Material 1. [file 12906_2026_5313_MOESM1_ESM.docx]

| **Section and Topic** | **Item #** | **Checklist item** | **Location where item is reported** |  |  |
| --- | --- | --- | --- | --- | --- |
| **TITLE** | | |  |  |  |
| Title | 1 | Identify the report as a systematic review. | L: 1–2, Title clearly identifies the study as a systematic review. |  |  |
| **ABSTRACT** | | |  |  |  |
| Abstract | 2 | See the PRISMA 2020 for Abstracts checklist. | L: 39–57, Abstract structured per PRISMA with objectives, methods, results, and conclusion. |  |  |
| **INTRODUCTION** | | |  |  |  |
| Rationale | 3 | Describe the rationale for the review in the context of existing knowledge. | L: 73–80, Introduction – Rationale and context linking yoga, stress, and AMH modulation. |  |  |
| Objectives | 4 | Provide an explicit statement of the objective(s) or question(s) the review addresses. | L: 84–95, Introduction – Explicit statement of the review objectives. |  |  |
| **METHODS** | | |  |  |  |
| Eligibility criteria | 5 | Specify the inclusion and exclusion criteria for the review and how studies were grouped for the syntheses. | L: 114–120, 2.2 Eligibility criteria – inclusion/exclusion per PICO Table 2. |  |  |
| Information sources | 6 | Specify all databases, registers, websites, organisations, reference lists and other sources searched or consulted to identify studies. Specify the date when each source was last searched or consulted. | L: 101–108, 2.1 Data sources – databases, websites, and last search date (21/01/2024). |  |  |
| Search strategy | 7 | Present the full search strategies for all databases, registers and websites, including any filters and limits used. | L: 101–104, Search strategy and filters described (PubMed, Cochrane, etc.). |  |  |
| Selection process | 8 | Specify the methods used to decide whether a study met the inclusion criteria of the review, including how many reviewers screened each record and each report retrieved, whether they worked independently, and if applicable, details of automation tools used in the process. | L: 107–113, Selection process – dual independent screening using Rayyan, conflicts resolved by VP. |  |  |
| Data collection process | 9 | Specify the methods used to collect data from reports, including how many reviewers collected data from each report, whether they worked independently, any processes for obtaining or confirming data from study investigators, and if applicable, details of automation tools used in the process. | L: 122–134, Data collection and confirmation process described. |  |  |
| Data items | 10a | List and define all outcomes for which data were sought. Specify whether all results that were compatible with each outcome domain in each study were sought (e.g. for all measures, time points, analyses), and if not, the methods used to decide which results to collect. | L: 155–162, Outcomes: AMH, androgen, insulin, FBG, BP, weight, etc. |  |  |
|  | 10b | List and define all other variables for which data were sought (e.g. participant and intervention characteristics, funding sources). Describe any assumptions made about any missing or unclear information. | L: 163–170, Other variables: participant characteristics, intervention duration, funding assumptions. |  |  |
| Study risk of bias assessment | 11 | Specify the methods used to assess risk of bias in the included studies, including details of the tool(s) used, how many reviewers assessed each study and whether they worked independently, and if applicable, details of automation tools used in the process. | L: 171–176, Risk of bias assessed using Cochrane RoB 2 tool. |  |  |
| Effect measures | 12 | Specify for each outcome the effect measure(s) (e.g. risk ratio, mean difference) used in the synthesis or presentation of results. | L: 177–182, Effect measures – mean difference (95% CI) for outcomes. |  |  |
| Synthesis methods | 13a | Describe the processes used to decide which studies were eligible for each synthesis (e.g. tabulating the study intervention characteristics and comparing against the planned groups for each synthesis (item #5)). | L: 183–190, Eligibility for synthesis and data organization described. |  |  |
|  | 13b | Describe any methods required to prepare the data for presentation or synthesis, such as handling of missing summary statistics, or data conversions. | L: 191–195, Unit conversions and data harmonization detailed. |  |  |
|  | 13c | Describe any methods used to tabulate or visually display results of individual studies and syntheses. | L: 196–204, Tables 3–5 and Figures 1–3 used for structured presentation. |  |  |
|  | 13d | Describe any methods used to synthesize results and provide a rationale for the choice(s). If meta-analysis was performed, describe the model(s), method(s) to identify the presence and extent of statistical heterogeneity, and software package(s) used. | L: 205–210, Narrative synthesis rationale provided (no meta-analysis due to limited data). |  |  |
|  | 13e | Describe any methods used to explore possible causes of heterogeneity among study results (e.g. subgroup analysis, meta-regression). | L: 309–316, Discussion – heterogeneity in intervention duration and content discussed. |  |  |
|  | 13f | Describe any sensitivity analyses conducted to assess robustness of the synthesized results. | Not applicable – no sensitivity analysis performed due to data limitations. |  |  |
| Reporting bias assessment | 14 | Describe any methods used to assess risk of bias due to missing results in a synthesis (arising from reporting biases). | L: 138–142, Publication bias discussed, qualitative RoB2 assessment used instead of funnel plot. |  |  |
| Certainty assessment | 15 | Describe any methods used to assess certainty (or confidence) in the body of evidence for an outcome. | L: 309–316, Certainty of evidence discussed via study quality and risk of bias. |  |  |
| **RESULTS** | | |  |  |  |
| Study selection | 16a | Describe the results of the search and selection process, from the number of records identified in the search to the number of studies included in the review, ideally using a flow diagram. | L: 217–222, Study selection results and PRISMA flow diagram (Figure 1). |  |  |
|  | 16b | Cite studies that might appear to meet the inclusion criteria, but which were excluded, and explain why they were excluded. | L: 223–231, Excluded studies and reasons described (n=25 excluded). |  |  |
| Study characteristics | 17 | Cite each included study and present its characteristics. | L: 232–256, Study characteristics detailed in Table 3. |  |  |
| Risk of bias in studies | 18 | Present assessments of risk of bias for each included study. | L: 257–262, Risk of bias summary in Figure 2. |  |  |
| Results of individual studies | 19 | For all outcomes, present, for each study: (a) summary statistics for each group (where appropriate) and (b) an effect estimate and its precision (e.g. confidence/credible interval), ideally using structured tables or plots. | L: 263–300, Results of individual studies presented in Tables 4–5. |  |  |
| Results of syntheses | 20a | For each synthesis, briefly summarise the characteristics and risk of bias among contributing studies. | L: 301–308, Summary of contributing studies’ risk of bias and characteristics. |  |  |
|  | 20b | Present results of all statistical syntheses conducted. If meta-analysis was done, present for each the summary estimate and its precision (e.g. confidence/credible interval) and measures of statistical heterogeneity. If comparing groups, describe the direction of the effect. | Not applicable – no statistical synthesis/meta-analysis performed. |  |  |
|  | 20c | Present results of all investigations of possible causes of heterogeneity among study results. | L: 309–316, Heterogeneity causes discussed (duration, frequency, protocol variation). |  |  |
|  | 20d | Present results of all sensitivity analyses conducted to assess the robustness of the synthesized results. | Not applicable – sensitivity analysis not performed. |  |  |
| Reporting biases | 21 | Present assessments of risk of bias due to missing results (arising from reporting biases) for each synthesis assessed. | L: 138–142, Risk of bias due to missing results discussed qualitatively. |  |  |
| Certainty of evidence | 22 | Present assessments of certainty (or confidence) in the body of evidence for each outcome assessed. | L: 309–316, Certainty of evidence and limitations discussed. |  |  |
| **DISCUSSION** | | |  |  |  |
| Discussion | 23a | Provide a general interpretation of the results in the context of other evidence. | L: 317–328, Discussion – interpretation of results compared with other evidence. |  |  |
|  | 23b | Discuss any limitations of the evidence included in the review. | L: 329–341, Discussion – limitations of included evidence (small n, heterogeneity). |  |  |
|  | 23c | Discuss any limitations of the review processes used. | L: 342–348, Discussion – limitations of review process and lack of meta-analysis. |  |  |
|  | 23d | Discuss implications of the results for practice, policy, and future research. | L: 349–360, Implications for practice and future research recommendations. |  |  |
| **OTHER INFORMATION** | | |  |  |  |
| Registration and protocol | 24a | Provide registration information for the review, including register name and registration number, or state that the review was not registered. | L: 39, PROSPERO registration ID: CRD42022342913 (10/07/2022). |  |  |
|  | 24b | Indicate where the review protocol can be accessed, or state that a protocol was not prepared. | L: 39, Protocol registered on PROSPERO, no separate protocol publication. |  |  |
|  | 24c | Describe and explain any amendments to information provided at registration or in the protocol. | Not applicable – no amendments to protocol. |  |  |
| Support | 25 | Describe sources of financial or non-financial support for the review, and the role of the funders or sponsors in the review. | L: 670–681, Funding and sponsor roles described. |  |  |
| Competing interests | 26 | Declare any competing interests of review authors. | L: 662–669, Competing interests declared as none. |  |  |
| Availability of data, code and other materials | 27 | Report which of the following are publicly available and where they can be found: template data collection forms; data extracted from included studies; data used for all analyses; analytic code; any other materials used in the review. | L: 654–661, Availability of data and materials described. |  |  |

*From:*  Page MJ, McKenzie JE, Bossuyt PM, Boutron I, Hoffmann TC, Mulrow CD, et al. The PRISMA 2020 statement: an updated guideline for reporting systematic reviews. BMJ 2021;372:n71. doi: 10.1136/bmj.n71. This work is licensed under CC BY 4.0. To view a copy of this license, visit <https://creativecommons.org/licenses/by/4.0/>
